# Transmission and scanning electron microscopy of *cutis rhomboidalis*^☆^^☆☆^

**DOI:** 10.1016/j.abd.2020.08.013

**Published:** 2021-03-15

**Authors:** Ângela Faistauer Torre, Hiram Larangeira de Almeida Junior, Valeria Magalhães Jorge, Antônia Larangeira de Almeida

**Affiliations:** a Universidade Católica de Pelotas, Pelotas, RS, Brazil; b Universidade Federal de Pelotas, Pelotas, RS, Brazil

**Keywords:** Elastic tissue, Microscopy, Microscopy, electron, scanning, Microscopy, electron, transmission

## Abstract

*Cutis rhomboidalis* nuchae was assessed in a 65-year-old patient. Optical microscopy showed basophilic agglomerations in the reticular dermis with decreased elastic fibers. Transmission electron microscopy showed elongated, curved and fragmented structures, and in their interior the presence of electron-dense lumps was reduced and irregular, similar to modified elastic fibers, whereas the collagen fibers had a normal aspect. Scanning electron microscopy showed deposits between the bundles of collagen, resembling pebbles or stones. These findings demonstrate that, at one stage of the disease, the collagen remains normal and the alterations are seen in the elastic tissue.

## Introduction

*Cutis rhomboidalis nuchae* (CRN) is a type of solar elastosis identified especially in patients with a history of chronic sun exposure while working. The skin of the posterolateral cervical region acquires the typical appearance of “leather”, with a yellowish and ridged surface.^1^ Skin alterations related to the aging process involve complex biological processes that basically consist of two mechanisms: chronology and sun exposure, the result of which are structural alterations in the dermis.^2^ Additionally, solar elastosis, including CRN, as markers of chronic exposure to sunlight, are a risk factor for premalignant and malignant skin lesions.^3^

**Figure 1:**
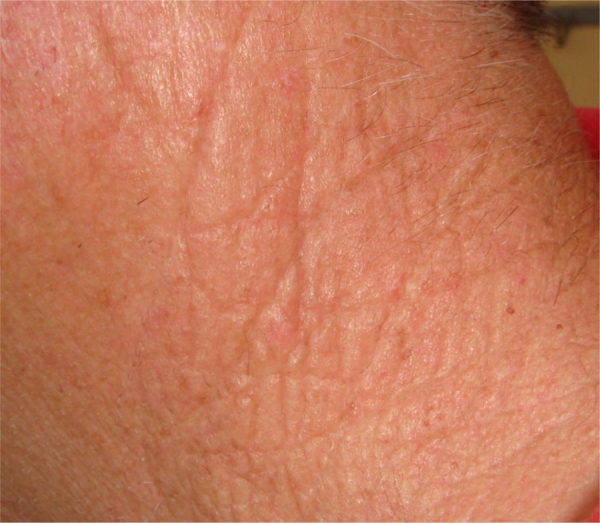
Clinical aspect on the left cervical region.

A small fragment of CRN skin was obtained from a 65-year-old patient with a history of chronic exposure to sunlight (Fig. 1). Part of the fragment was processed for optical microscopy and another part was prepared for Transmission Electron Microscopy (TEM) analysis, with the ultrathin sections directed to the high reticular dermis; a third part of the skin was dehydrated and metalized, to examine the surface of the dermis with Scanning Electron Microscopy (SEM).

## Results

**Figure 2:**
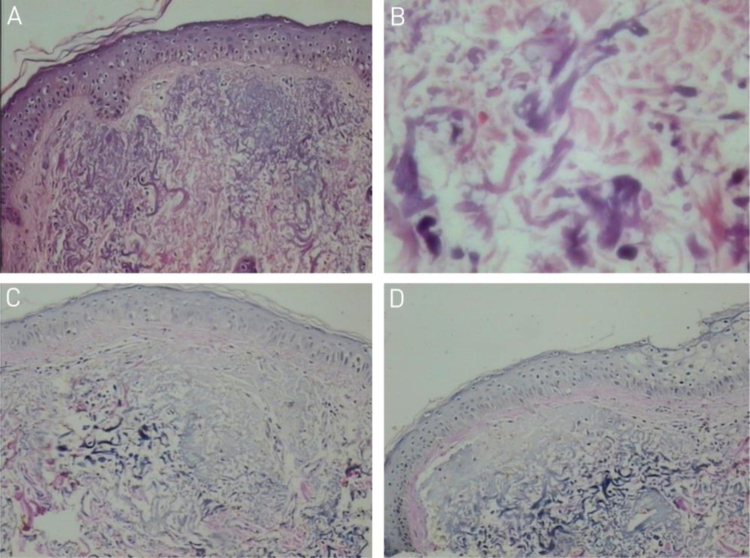
Optical microscopy: (A) basophilic alterations in the superficial dermis (Hematoxylin & eosin ×150). (B) Detail of basophilic alterations with irregular and fragmented material (Hematoxylin & eosin, ×400). (C and D) Reduced elastic fibers in the basophilic areas (Verhoeff, ×150).

Optical microscopy with hematoxylin and eosin staining showed flattening of the epidermis, with basophilic clumps in the reticular dermis (Fig. 2A); on high power the basophilic structures had an irregular outline and were fragmented (Fig. 2B). Verhoeff's staining for elastic fibers showed a decrease in basophilic areas (Fig. 2C and D).

**Figure 3:**
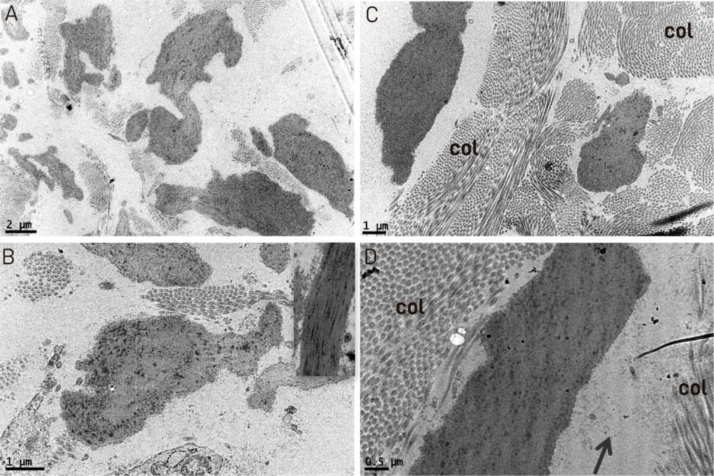
Transmission electron microscopy: (A) fragmented and irregular elastic fibers (×18,000). (B) detail of an irregular elastic fiber with modification in the distribution of dark spots inside it, inset showing normal fiber for comparison (×20,000). (C) normal collagen fibers (col) (×12,000). (D) Detail of an altered elastic fiber with an irregular interior, deposition of amorphous material (arrow) and normal collagen (col) in cross-sectional and longitudinal sections, with normal periodicity in the lower right quadrant (×25,000).

Transmission electron microscopy of the reticular dermis showed findings that were similar to those observed on high power in optical microscopy, with the presence of elongated, curved, and fragmented structures (Fig. 3A). Observing the interior of the elongated and fragmented structures, the presence of lumps of dense electron material inside them was reduced and irregular (Fig. 3B, C and D), similar to modified elastic fibers, whose matrix under normal conditions shows a uniform distribution of these lumps (Fig. 3B). The collagen fibers showed no alterations, maintaining the typical fiber periodicity in ultrastructure (Fig. 3C and D), while deposition of amorphous material was observed in some areas (Fig. 3C).

**Figure 4:**
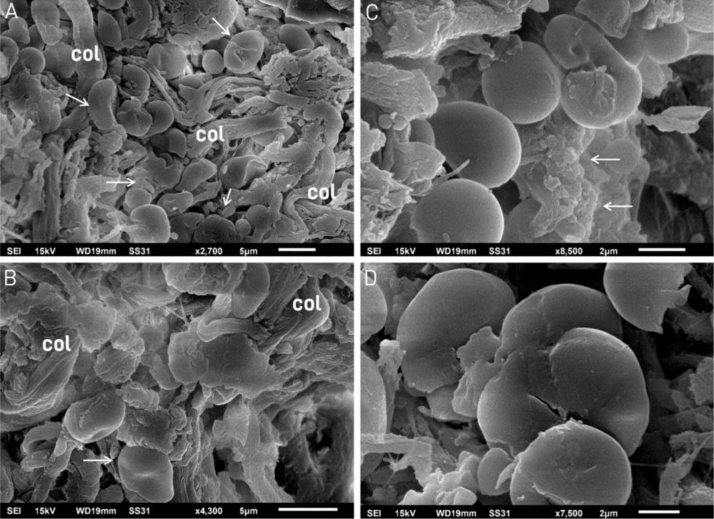
Scanning electron microscopy: (A and B) fragmented tissue resembling pebbles or stones (arrows) between the collagen fibers (col). (×2,700 and ×4,300). (C) Detail of four structures resembling pebbles or stones; the upper one is curved, similar to Fig. 3A, and a granular deposit is seen on the right (arrows) (×8,500). (D) Detail of the deposits resembling pebbles or stones (×7,500).

Scanning electron microscopy of the dermis surface showed deposits between the collagen bundles (Fig. 4A and B) and on high power the deposits appeared rounded resembling pebbles or stones and irregular contour structures (Fig. 4C and D). In some areas, deposition of granular material was observed (Fig. 4C).

## Discussion

CRN is a frequent finding in physical examinations and a marker of chronic photo exposure, being correlated with the prevalence of malignant and premalignant lesions.^3^, ^4^

Our optical microscopy findings showed a basophilic deposition in the upper dermis, called solar elastosis, and basophilic degeneration of collagen.^5^ In these areas, after elastic fiber staining, it was possible to see a decrease in these fibers.

Transmission electron microscopy, where tissue sections are examined, allowed us to observe that the collagen fibers were normal in appearance, unlike the elastic fibers, which showed an irregular contour and were fragmented, demonstrating that at least during one phase of the disease, the collagen remains normal, in agreement with previous publications.^5^, ^6^ The observed deposition of amorphous material could be glycosaminoglycans, described in biochemical studies.^7^

The three-dimensional findings with scanning electron microscopy also showed fragmented tissue in the dermis together with collagen bundles. These findings of deposits resembling small stones or pebbles have already been described in other dermal conditions, demonstrating the superiority of TEM in the analysis of elastic fibers, with a better demonstration of the internal changes in fibers in tissue sections.^8^, ^9^ These findings are in agreement with a previous publication, which showed that initial lesions have deposits in more affected areas, large amorphous masses, and that the elastotic material is comprised of elastic fibers.^10^ The deposition of amorphous material associated with elastic fiber degeneration has been reported, as well as preserved collagen fibers and bundle disorganization.^11^

Considering these results, the histological expression basophilic degeneration of collagen should be replaced by basophilic dermal degeneration, while the expression, solar elastosis, should be utilized as a clinical term and not as a histological one.

## Financial support

None declared.

## Authors' contributions

Ângela Faistauer Torre: Approval of the final version of the manuscript; drafting and editing of the manuscript; critical review of the literature; critical review of the manuscript.

Hiram de Almeida Jr: Approval of the final version of the manuscript; drafting and editing of the manuscript; critical review of the literature; critical review of the manuscript.

Valeria Magalhães Jorge: Approval of the final version of the manuscript; drafting and editing of the manuscript; critical review of the literature; critical review of the manuscript.

Antônia Larangeira de Almeida: Approval of the final version of the manuscript; drafting and editing of the manuscript; critical review of the literature; critical review of the manuscript.

## Conflicts of interest

None declared.
